# Measurement of transverse forces between the first and second metatarsals: a cadaveric study

**DOI:** 10.1186/s13018-016-0459-x

**Published:** 2016-10-17

**Authors:** Viktor Feldman, Meir Nyska, Niv Marom, Omer Slavin, Yaron S. Brin, Uri Farkash, Ezequiel Palmanovich

**Affiliations:** 1 Orthopedic Department, Meir General Hospital, Sapir Medical Center, Kfar Saba, Israel; 2 Sackler Faculty of Medicine, Tel Aviv University, Tel Aviv, Israel

**Keywords:** Hallux valgus, CyclaPlex™, Mini TightRope®, Intermetatarsal forces, Foot osteotomy

## Abstract

**Background:**

This study was designed to measure transverse forces between the 1st and 2nd metatarsals after reducing the intermetatarsal angle (IMA) in normal and hallux valgus (HV) feet, during non weight-bearing and weight-bearing phases of gait.

**Methods:**

Four cadaver feet, three normal and one with hallux valgus, were used. A new suture button device (CyclaPlex™) composed of screw-type buttons connected with a wire was implanted at the mid-shaft of the 1st and 2nd metatarsals of all the feet. IMA was reduced using a tensioning device to pull the wire which was secured laterally at the 1st metatarsal. The 1st metatarsal was pulled laterally towards the 2nd metatarsal until an IMA of about 6° was achieved. The amount of force applied at this point was registered on the force indicator. Each foot attached to the tensioning device was placed in a special construct loaded with weights equal to the original body weight of the donor and positioned at 15° tilt (simulating propulsion phase of the gait cycle). The intermetatarsal force under load indicated on the tensioning device was recorded.

**Results:**

The average recorded transverse intermetatarsal force was 28.5 N (SD 4.2 N) during non weight-bearing phase; the mean increase in the measured force at weight-bearing and 15° tilt was 6 N (SD 2.6 N).

**Conclusions:**

We measured the transverse forces between the 1st and 2nd metatarsals with the use of a suture button device (CyclaPlex™). The data obtained from the measurements will provide a better understanding of foot biomechanics and may therefore also facilitate the development of new devices designed to decrease IMA in HV surgery.

## Background

Hallux valgus (HV), one of the most common disorders of the foot causing pain and disability, is often treated by osteotomies. Osteotomies are invasive procedures used to correct 1st metatarsal deformity; however, osteotomies may lead to numerous complications and patient morbidity. Recently, new suture button devices have been used to avoid bone osteotomy. Direct measurement of the transverse forces between the 1st and 2nd metatarsals can advance the design of such devices.

The main deformities in HV are increased valgus of the 1st toe (HVA) and 1st metatarsal primus varus, resulting in an increased angle between the 1st and 2nd metatarsals (IMA). IMA is considered abnormal when values are greater than 9° [1]. Heredity is likely to be a major predisposing factor with up to 68 % of patients showing familial tendency [2, 3]. Footwear may be a contributing factor in a foot that is predisposed to HV [4]. The pathogenesis of HV has been well described by Stephens [5] and is beyond the scope of this article.

Osteotomy, generally considered to be a conservative procedure, is the preferred method of treatment. Based on severity of the deformity, proximal and/or distal osteotomies are recommended [6]. The goal in correcting the deformity is to reposition the 1st metatarsal to reduce the IMA. More than 130 surgical procedures to correct this deformity have been described in the literature.

**Fig. 1:**
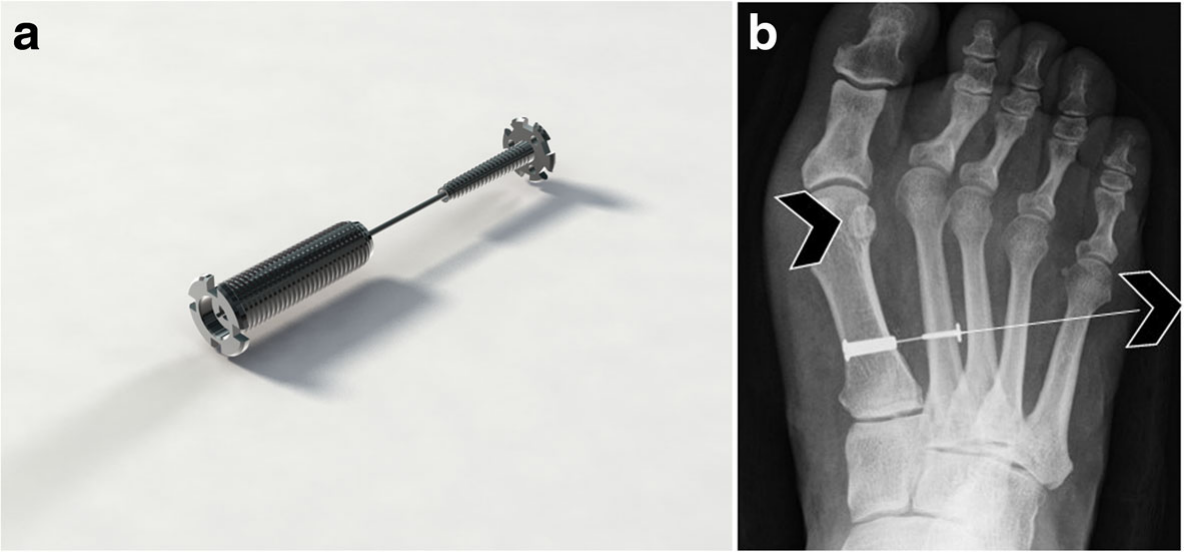
**a** CyclaPlex™ implant device. The device is made up of two screw-type buttons (anchors) and a connecting wire between them. The medial button (*on the left end*) is inserted into the first metatarsal. The wire protrudes from the lateral button (*on the right*). **b** The 2nd metatarsal serves as an anchor. The 1st metatarsal is pulled laterally by pulling the wire protruding from the 2nd metatarsal. The implant device remains in the foot. The *arrowheads* indicate the direction in which both the wire and the 1st MT are being pulled

A new suture button technique (CyclaPlex™, Cycla Orthopedics Ltd., Israel) has been developed. It is intended to reposition the 1st metatarsal and reduce the IMA without 1st metatarsal osteotomy [7]. The MiniTightRope® (Arthrex Inc, Naples, USA) is the leading suture button implant on the market [8]. Published clinical studies have shown that the MiniTightRope® provides generally good results; however, there are indications of post-operative complications such as 2nd metatarsal stress fractures and recurrence due to implant failure [9, 10]. The new suture button implant device has been developed to realign the 1st metatarsal bone (reduce the IMA) without performing the traditional 1st metatarsal osteotomy. The device comprises two screw-type buttons connected with a metal wire (Fig. 1a, b). In order to design a durable yet compact device, it is imperative to understand the transverse forces acting between the 1st and 2nd metatarsals as these forces must be overcome in order to reduce IMA and maintain its position for an extended period. Information about these forces will improve our understanding of 2nd metatarsal stress fractures and post-operative increase of IMA.

Up to now, studies have been conducted to measure these forces indirectly by extrapolating an estimate from the peak vertical ground reaction force under the 1st or 2nd metatarsal head during forefoot loading. Ground reaction forces were recorded, and multiple dimensions and angles of the metatarsal bones were measured. From these measurements, forces acting on the metatarsals were indirectly estimated [11–13]. To the best of our knowledge, transverse forces between the 1st and 2nd metatarsals have not been directly measured.

The purpose of this study was to directly measure the transverse forces between the 1st and 2nd metatarsals after reduction of IMA in normal and in hallux valgus (HV) feet at two phases of gait: non weight-bearing and full weight-bearing phases.

This is the first time direct measurement of transverse forces between the 1st and 2nd metatarsals in the foot has been achieved.

## Methods

### Materials

Four cadaveric specimens, mid-tibia to toes, were utilized for this study. Three normal feet (cases 1, 2, and 3) and one with HV deformity (case 4) were used. There were no additional pathologies. Specimens were taken from two male and two female donors, with a reported age at death ranging from 70 to 80 years. The average weight of the four donors was 60.7 kg (SD 14.5 kg) as shown in Table 1. All the specimens were sealed in airtight plastic bags and maintained in a frost-free freezer (−20 °C). The specimens were defrosted at room temperature (+20 °C) 1 day before the procedure.

**Table 1:** Specimen characteristics

Case	Gender	Age	Weight (kg)	Height (cm)	Initial IMA (deg.)	HVA (deg.)
1	M	74	56	177	6	19
2	M	70	77	167	10	27
3	F	74	49	165	10	17
Average		72.7	60.7	169.7	8.7	21
4	F	80	59	152	15	35
Total average		74.5	60.25	165	10.25	24.5

The study was performed in a surgery room designed for cadaver dissections. X-rays were taken during the implantation process using a portable digital X-ray imager (Girth Ultra Light, Panel: Vidisco RayzorX Pro, software: Xbit Pro).

**Fig. 2:**
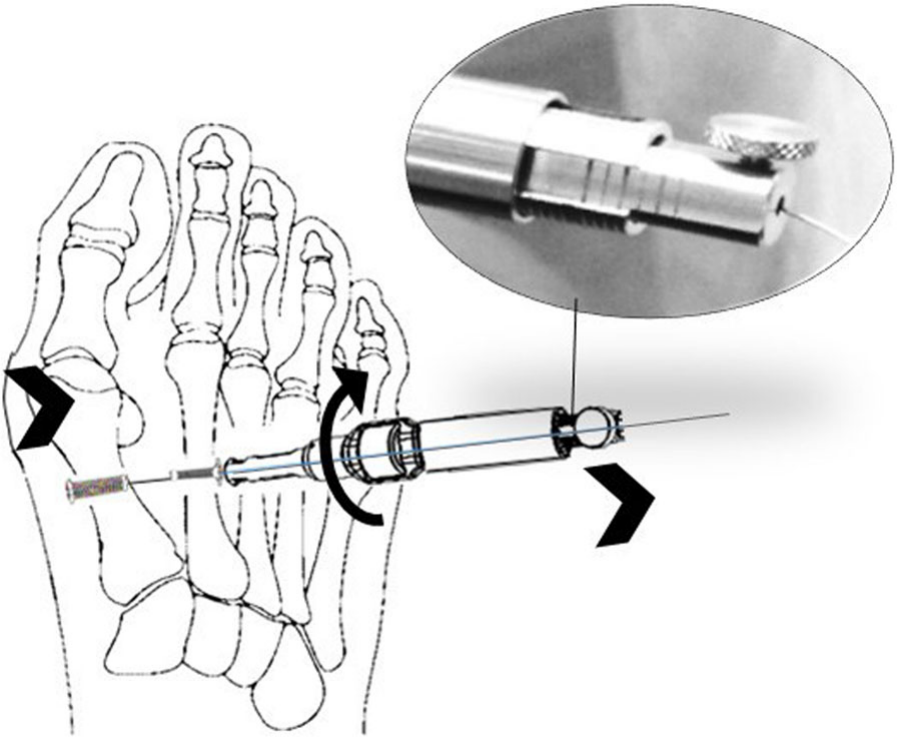
Tension instrument force indicator shows the amount of force applied during tensioning. Tension instrument is attached to the wire on the 2nd metatarsal; the force indicator (seen in the *circle*) shows the amount of force applied during tensioning by turning the knob (see *semicircular arrow*)

Four CyclaPlex™ implant devices and implantation instruments were provided by Cycla Orthopedics Ltd., Israel. The instrument comprises two components: an implantation device made up of two screw-type buttons (same head size) connected with a metal wire (Fig. 1a, b) and a unique tensioning and measuring device (Fig. 2). The tensioning device is connected to the wire projecting from the lateral button. By rotating the knob on the tensioning device (Fig. 2), the wire is pulled laterally, pulling the 1st metatarsal towards the 2nd metatarsal. The measure of force applied is indicated by markings in increments of 10 N (0–50 N) on the device.

**Fig. 3:**
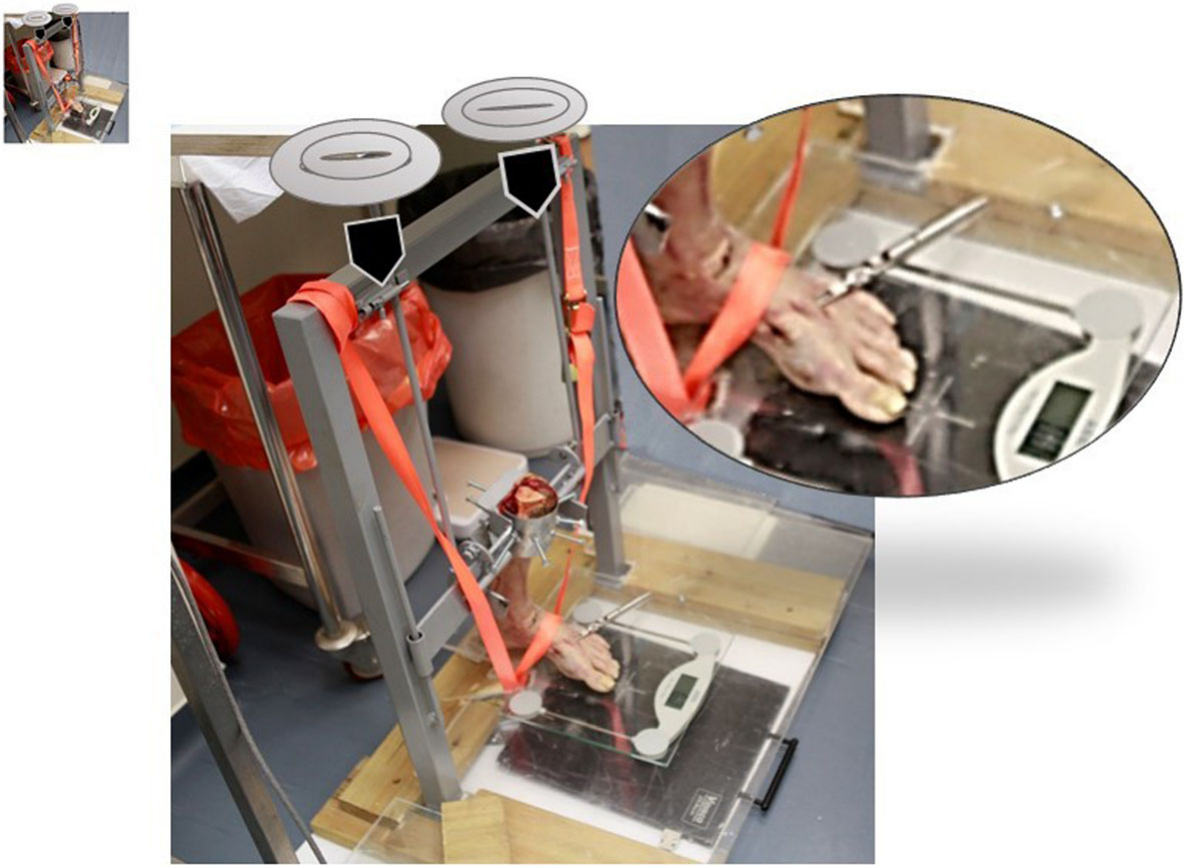
The forefoot was mounted on a scale at 15° tilt and restrained by straps. Weights were added until the scale showed the desired weight (original weight of the specimen donor). *Arrows* represent the weights which are added and placed on the top of the construct

A special construct was designed to hold the leg in a vertical position. The leg was fastened in the construct and mounted on a scale with the foot tilted at 15° of plantar flexion utilizing metal restraints and straps (Fig. 3). Weights were placed on top of the frame of the construct to match the original body weight of the specimen.

### Methods

To minimize variability, all procedures were performed by the same senior foot and ankle surgeon with the assistance of an orthopedic resident. The device was implanted percutaneously in the metatarsal mid-shafts. No lateral soft tissue release was performed. A longitudinal skin incision of 1 cm dorsally at the level of the 2nd metatarsal shaft and another 1-cm longitudinal incision at the medial aspect, over the 1st metatarsal shaft, were made. With the aid of a drill guide, a 1.5-mm opening was then drilled through both metatarsals using a battery-charged drill (Konisha, Japan). The 1st metatarsal opening was then widened using a 3.4-mm cannulated drill. A dedicated screw driver was used to screw a metal button into the hole prepared in the 1st metatarsal. An 18-cm-long cobalt chrome wire was passed through the metal button in the 1st metatarsal and then through the drilled hole in the 2nd metatarsal. The 2nd button (total diameter 1.6 mm, lumen diameter 0.9 mm) was threaded onto the lateral end of the wire and then screwed into the drill hole of the 2nd metatarsal using the same screw driver. The CyclaPlex™ device was implanted close to the middle of the 1st metatarsal shaft, avg. location at 38.8 % (SD 6.3 %) of the length of the metatarsal, measured from the proximal end.

After implantation of the device in the bones, the tensioning device was threaded onto the projecting lateral edge of the wire. The tensioning device head was positioned on the 2nd metatarsal button head on the lateral side of the bone.

Tensioning was performed by rotating the tensioning knob clockwise, thereby gradually pulling the wire outward. This resulted in pulling the 1st metatarsal laterally towards the 2nd metatarsal bone (Fig. 1b). Tensioning was performed until an IMA of about 6° was reached (except in case 1 where the initial IMA was 6°; in this case, the IMA was reduced to 2.5°). The force required to reduce the IMA as described was recorded for each specimen.

X-ray images of the foot were taken before, during, and at the end of tensioning. The device was maintained in a tensioned state in the foot for the next stage.

A second (weight-bearing) stage of the study was conducted. At this stage, the foot was mounted on a weight scale at 15° tilt and restrained with straps. Weights were added to the top of the construct until the original weight of the specimen donor was reached, thereby simulating the exact physiological load which each foot would bear (Fig. 3). The transverse intermetatarsal forces were measured in this loaded position, and the differences between the forces at unloaded and loaded states were calculated.

The measured intermetatarsal forces at the loaded state, and the calculated increase in the forces in the unloaded compared to the loaded state, apply to the three normal feet only.

### Statistical analysis

Descriptive statistics, mean, and standard deviation were calculated (MS Excel) for the following variables: implant location, initial IMA, final IMA, IMA reduction, intermetatarsal tension of loaded and unloaded feet, tension for IMA reduction per degree, and the tension difference between the unloaded and loaded states of the foot.

## Results

Specimen characteristics are summarized in Table 1.

IMA and measured intermetatarsal forces are summarized in Table 2.

**Table 2:** Results

Case	Implant location: distal position relative to 1st bone length (%)	Initial IMA^a^ (deg.)	Final IMA^a^ (deg.)	IMA reduction (deg.)	Tension unloaded (N)	Tension per 1° reduction (N)	Tension loaded (N)	Tension increase (N)
1	40	6	2.5	3.5	28	8.0	32	4
2	40	10	5.5	4.5	30	6.7	35	5
3	30	10	6	4	23	5.5	32	9
4	45	15	7	8	33	4.1	–	–
Avg. (±SD) of cases 1–3	36.7 (5.8)	8.7 (2.3)	4.7 (1.9)	4 (0.5)	27 (3.6)	6.8 (1.1)	33 (1.7)	6 (2.6)
Avg. (±SD) of cases 1–4	38.8 (6.3)	10.3 (3.7)	5.3 (1.9)	5.3 (1.9)	28.5 (4.2)	6.1 (1.6)	–	–

^a^IMA was measured on X-ray images using a goniometer

Three feet had normal IMA (avg. 8.7°, SD 2.3), and one foot had HV deformity with IMA of 15°. At the end of the tensioning process, the IMA of the feet was reduced by an average of 5.3° (SD 1.9). The transverse intermetatarsal forces measured during the study were between 23 and 33 N (avg. 28.5 N, SD 4.2). The calculated force required to reduce the IMA by 1° was between 5.5–8 N in the normal feet (avg. 6.8 N, SD 1.1) and 4.1 N in the HV foot. When the feet were loaded to the original body weight and 15° tilt, the intermetatarsal forces measured were between 32 and 35 N (avg. 33 N, SD 1.7). The calculated increase in intermetatarsal tension was between 4 and 9 N (avg. 6 N, SD 2.6). We were unable to measure tension in the loaded state on the HV specimen due to technical difficulties with the X-ray machine at this point. This prevented any further measurement of the HV foot for the trial.

## Discussion

Hallux valgus is a frequent cause of foot pain and disability, with an estimated 500,000 operations performed in the USA each year for deformity correction [14]. The number of techniques described for the treatment of HV indicates that no single operation is perfect, and no one particular operation addresses all cases [2]. The standard options for surgical treatment of HV include distal osteotomies, proximal osteotomies, or combined distal and proximal osteotomies. The most common complications include failure, recurrence of deformity, malunion, nonunion, hallux varus, 2nd toe transfer metatarsalgia, and avascular necrosis [2, 15–17].

Up to a third of treated patients may be dissatisfied with the outcome of surgery [18]. Havlícek V. et al. reported that only 60 % of the patients who underwent McBride’s procedure (45 operations) were satisfied [19]. Less invasive surgical techniques are continuously being developed to avoid complications and minimize post-operative morbidity [20]. In order to perform a successful implantation of such devices and minimize hardware failure and post-operative complications, knowledge and understanding of the transverse intermetatarsal forces in non weight-bearing and weight-bearing states is essential.

The results of this study on the transverse forces between the 1st and 2nd metatarsals show that in order to correct the IMA to about 5° in normal feet, an average force of only about 33 N is needed. Despite the small sample, we found that the forces measured in the normal feet and in the HV foot in the unloaded state were similar. Contrary to our expectations, the increased force between the metatarsals during propulsion at full weight-bearing, compared to the foot at the resting state, was only slightly higher (increase of 4–9 N). We anticipate that over time, the intermetatarsal force will decrease even further as tissue relaxation takes place. The force required to reduce the IMA by 1° is less in the HV foot than in normal feet. This may be due to the fact that the 1st tarsometatarsal joint (TMTJ) in HV patients is hypermobile compared to normal feet.

When the IMA is smaller, greater force must be applied in order to achieve 1° of IMA reduction. This is due to resistance of soft tissues which must be overcome.

To the best of our knowledge, this is the first study where intermetatarsal forces have been measured directly; thus, for the first time, we are able to examine the magnitude of the force between the 1st and 2nd metatarsals.

The aim in correcting the deformity in HV is to decrease the IMA. This is usually performed by osteotomy; however, as mentioned previously, correction of HV and reduction of IMA with less invasive surgical techniques are continuously being developed as they allow for faster recovery. In order to develop suture button devices, compression forces applied between the 1st and 2nd metatarsals must decrease IMA; however, the amount of force required to reduce the angle is unknown. We believe that the information obtained from our study will increase understanding of the prevailing intermetatarsal forces in the foot and will therefore provide important information for development of devices designed to decrease the intermetatarsal angle in HV patients. When forces between the 1st and 2nd metatarsals during foot loading are better understood, surgical techniques and implants can be designed to reliably reduce and maintain the IMA without resorting to fusion of the 1st metatarsal cuneiform joint in the treatment of hallux valgus. Recognition of the amount of force will help develop devices which are small enough and require a minimal invasive approach but are strong and reliable enough to withstand the force.

This is a pilot study and its limitations include the following: the small sample size used. Further cadaveric trials should be conducted to increase the statistical validity. Another limitation is the difficulty of applying the results of an adult cadaver model to living patients who have greater biological plasticity. In addition, cadaveric specimens might not reliably simulate the weight-bearing state of a living patient. The precision of the CyclaPlex™ device is limited to 10-N increments. A more precise measuring device (presently unavailable) would provide more accurate force measurement.

It is clear that confirmation of the data presented must await additional clinical trials.

## Conclusions

The CyclaPlex™ implant device was used to directly measure the forces between the 1st and 2nd metatarsals. Information obtained from this study may serve as a basis for future development of devices designed to reliably reduce and maintain the IMA without resorting to fusion of the 1st metatarsal cuneiform joint in the treatment of hallux valgus. Information regarding the optimal force required to reduce the IMA should aid in the development of devices which are small enough to require a minimal invasive approach yet are strong enough to withstand the force applied. It is clear that confirmation of the data presented must await future clinical trials.

## Abbreviations

HV: Hallux valgus
IMA: Intermetatarsal angle
N: Newtons
SD: Standard deviation
